# Stroke Rehabilitation for Falls and Risk of Falls in Southeast Asia: A Scoping Review With Stakeholders' Consultation

**DOI:** 10.3389/fpubh.2021.611793

**Published:** 2021-03-03

**Authors:** Husna Ahmad Ainuddin, Muhammad Hibatullah Romli, Tengku Aizan Hamid, Mazatulfazura S. F. Salim, Lynette Mackenzie

**Affiliations:** ^1^Center of Occupational Therapy Studies, Faculty of Health Sciences, Universiti Teknologi MARA Selangor, Selangor, Malaysia; ^2^Department of Rehabilitation Medicine, Faculty of Medicine and Health Sciences, Universiti Putra Malaysia, Serdang, Malaysia; ^3^Malaysian Research Institute on Ageing, Universiti Putra Malaysia, Serdang, Malaysia; ^4^Discipline of Occupational Therapy, Faculty of Medicine and Health, School of Health Sciences, University of Sydney, Sydney, NSW, Australia

**Keywords:** aged, cerebrovascular accident, falls, rehabilitation, developing countries

## Abstract

**Background:** Research on rehabilitation for falls after stroke is warranted. However, published evidence on fall interventions with stroke survivors is limited and these are mainly international studies that may be less relevant for Southeast Asia.

**Objective:** This review aims to systematically identify literature related to stroke rehabilitation for falls and risk of falls in Southeast Asia.

**Methods:** A scoping review with stakeholders' consultation was implemented. An electronic search was conducted up to December 2020 on 4 databases (Medline, CINAHL, Scopus, ASEAN Citation Index). Only original studies conducted in Southeast Asia were selected.

**Results:** The initial search yielded 3,112 articles, however, only 26 were selected in the final analysis. Most of the articles focused on physical rehabilitation and implemented conventional therapies. While the literature may reflect practice in Southeast Asia, stakeholders perceived that the literature was inadequate to show true practice, was not informative and missed several aspects such as functional, cognitive, and psychological interventions in managing falls. Individual-centric interventions dominated the review while community-based and environmental-focused studies were limited. Majority of the articles were written by physiotherapists while others were from physicians, occupational therapists, and an engineer but few from other healthcare practitioners (i.e., speech therapists, psychologists) or disciplines interested in falls.

**Conclusions:** Falls prevention among stroke survivors has received a lack of attention and is perceived as an indirect goal in stroke rehabilitation in Southeast Asia. More innovative research adopted from falls research with older people is needed to advance falls prevention and intervention practice with stroke survivors.

## Introduction

Southeast Asia is one of the most populous regions in the world. It consists of low, middle, and high-income countries namely Brunei, Cambodia, East Timor, Indonesia, Laos, Malaysia, Myanmar, the Philippines, Singapore, Thailand, and Vietnam (1). With a total population of almost 700 million, the region is rich with different ethnicities and cultures. However, it also shares similarities in terms of dietary, climate and lifestyle (1, 2). Stroke is one of the top non-communicable diseases worldwide and in Asia (3). The highest prevalence rates of both ischemic and haemorrhagic stroke occur in high-income regions of the Asia Pacific, North America, East and Southeast Asia, in those aged 50–64 years (4). The prevalence of stroke in Southeast Asia countries per 1000 people is 8.0 in Indonesia (5), 9.0 in the Philippines (6), 36.5 (for >50 years) in Singapore (7), 18.8 (for >45 years) in Thailand (8), 6.1 in Vietnam (9) and 7.0 in Malaysia (10) respectively. Stroke is the primary cause of physical impairment and disabilities in adulthood making it a major public health problem (11–13).

Studies have established the association between stroke and falls (14, 15). The consequences of falls after stroke are devastating as they could lead to reduced social participation, fear of falling, traumatic brain injury, fractures, deficits in functioning, morbidity, and even mortality (16–19). International studies reported that the prevalence of falls among stroke survivors within the first 6 months after discharge is between 36 to 73% and fall rates remain high between 40 to 58% 1 year after stroke (20–25). However, the prevalence of falls among the stroke population in Southeast Asia has not yet been determined. Furthermore, 61% of falls occurred in the first 2 months after discharge from rehabilitation and returning home after stroke (26). Most stroke survivors fall inside their home (27–29) and it was reported that walking and transfers were the most frequent activities at the time of a fall (16). Mackintosh et al. (26) also reported that less than a quarter of fallers sought health professional advice after a fall and possible reasons for this include the perception that falls are not preventable and occur because of the impairments after a stroke or an individual's age. This finding suggests there is a need to instigate more intensive falls and injury prevention strategies in the first months after discharge from rehabilitation.

Falls in stroke survivors are usually attributed to a combination of factors that may or may not be related to stroke, and stroke is just one of the many significant comorbidities affecting older adults (30). Several risk factors for falls among stroke survivors have been identified. The key risk factors for fallers among the stroke population are impaired mobility, reduced balance, use of sedative/psychotropic medications, disability in self-care, depression, cognitive impairment, and history of falls (31). Previous literature on falls in older adults (32, 33) and the stroke population (31, 34) proposed a classification of risk factors of falls into the following domains: sociodemographic, sensorimotor, cognitive, psychosocial, medical, balance and mobility, and self-care (31). Several studies have concluded that the risk factors identified for falls in stroke are similar to those of falls in the general older population (30, 35, 36). However, compared with studies among older people (37–39), research in falls and stroke is limited, particularly in aspects other than physical issues, either as a risk factor or intervention. These findings suggest a need to establish holistic and tailored falls and injury prevention strategies as an integral part of each person's stroke rehabilitation plan. Thus, by understanding falls in stroke, preventing and intervening falls needs to become a priority for stroke survivors.

Rehabilitation is a goal-oriented process that assists a person with disabilities to achieve an optimal emotional, physical, cognitive, social, and functional level (40). The professionals involved include rehabilitation physicians, occupational therapists, physiotherapists, speech therapists, rehabilitation nurses, and medical social workers. In general, the rehabilitation process can occur either as an inpatient, outpatient, in the community, and in home-based settings. Rehabilitation was found to benefit stroke and falls in general (37, 41, 42). These rehabilitation programs must be carefully evaluated based on understanding the risk factors contributing to falls (43). Stroke rehabilitation for fall prevention aims to correct factors that have contributed to the fall, assist recovery from any complications and restore confidence and activity. This process includes a falls risk assessment including fear of falling, a comprehensive home assessment to reduce falling hazards, and multifactorial interventions in the community (44). One systematic review identified several interventions for falls, including exercise, pre-discharged home visits, the use of an assistive device, and transcranial direct current stimulation. However, only single exercise interventions were found might reduce the rate of falls among stroke survivors (45).

Published evidence on interventions for stroke and falls is limited and are mainly from Western-influenced and developed countries. This signifies that the findings on stroke and falls practice from those countries' contexts may become less relevant for application in Southeast Asia (40, 41). Studies on falls and community-dwelling older people gathered from a specific region benefit the region by identifying unique findings, practicing gaps, and providing a better understanding of the field for the region (46). While stroke and falls are also a major concern in Southeast Asia, published literature in this region remains limited and difficult to access.

A scoping review's general purpose is to map key concepts that underpin a research area, especially an area that is complex or understudied (47). Thus, this review, guided by the JBI guideline for evidence synthesis (48), aims to comprehensively identify evidence on stroke rehabilitation for falls and risk of falls in Southeast Asia. The framework consists of seven consecutive stages, which include (i) developing the review question, (ii) defining inclusion and exclusion criteria, (iii) conducting a search strategy, (iv) evidence screening and study selection, (v) data extraction, (vi) data analysis, and (vii) presentation of results. Each stage is discussed in further detail below, and the Preferred Reporting Items for Systematic Reviews and Meta-analysis extension for scoping reviews (PRISMA-ScR) (49) (Supplementary Table 1) was adopted as a guideline for the report of the scoping review.

## Methodology

### Review Question

The study adopted the Population-Concept-Context (PCC) framework (48) to determine the research question's extent. The population is adult stroke patients, and the concepts are rehabilitation for falls and risk of falls after stroke. The context of this study focused on the Southeast Asia region. This scoping review was developed based on the question “What is the extent of published literature on rehabilitation for falls and risk of falls after stroke in Southeast Asia?”

### Defining Inclusion and Exclusion Criteria

The criteria are detailed according to the PCC framework as the following:

#### Participants

This scoping review considered research studies that focused on stroke survivors, caregivers and healthcare professionals aged 21 years old and above. Stroke survivors were defined according to diagnostic criteria of the American Heart Association/American Stroke Association (50). All professionals were part of the management team for stroke patients, including rehabilitation physicians, physicians, medical social officers, occupational therapists, speech therapists, physiotherapists, engineers, and volunteers.

#### Concept

This review considered all research studies that specifically addressed stroke rehabilitation for falls or risk of falls. Research that did not provide stroke rehabilitation services was excluded.

#### Context

All studies need to be conducted in Southeast Asia, and interventions could be implemented either in the hospital or in the community (e.g., patients' homes). However, residential and institutionalized stroke patients were excluded from the analysis; stroke patients in institutions have severe disabilities, and the main aim of the care in these institutions is nursing rather than rehabilitative care.

#### Types of Evidence Source

Studies included in this review were all types of original or primary research studies including interventional studies (randomized controlled trials, quasi-experiments, single-group pre-post), observational studies (cross-sectional, cohort, longitudinal, case series, and case reports) and qualitative studies related to rehabilitation. A scoping review aims to provide a map of understanding about a scenario; thus, it will consider various designs of original studies including articles of lower evidence such as qualitative studies and case reports (48). All full-text articles included must be in English. Gray literature (e.g., thesis, dissertation), conference abstracts, guidelines, training, process evaluations (no outcome data), non-research article (i.e., editorial, letter, commentary) and secondary data analyses (books, government reports) were excluded from the review. No restrictions were imposed on the date of the study or the study design.

### Search Strategy

The search strategy was used to locate published studies. First, keywords derived from previous systematic reviews and other reviews (30, 31, 45) were listed to get an idea of the common keywords utilized for the topic. Then, based on the PCC framework, the authors developed the main keywords for each category. Synonyms for each keyword were searched via the internet and discussions were conducted between 2 authors to select and finalize the most relevant keywords for use. The finalized keywords utilized are as the following: a combination of “stroke” and related terminology (i.e., cerebrovascular accident, CVA), “rehabilitation” (including occupational therapy, physiotherapy, speech therapy), “falls” (including falls intervention and prevention) and “Southeast Asia” (including each name of the country members). Boolean operators, parenthesis, exact and wildcards were used whenever appropriate.

Specifically, the search strings used were (“cerebrovascular accident” OR “CVA” OR “stroke”) AND (“fall^*^” OR “fall^*^ prevention” OR “fall^*^ intervention” OR “accidental fall^*^” OR “risk of fall^*^” OR “fall^*^ predictor^*^” OR “prevalence of fall^*^”) AND (“rehabilitation” OR “occupational therap^*^” OR “physiotherapy^*^” OR “physical therap^*^” OR “speech language patholog^*^” OR “speech therap^*^”) AND (“Southeast Asia” OR “Malaysia” OR “Singapore” OR “Thailand” OR “Brunei” OR “Indonesia” OR “East Timor” OR “Cambodia” OR “Myanmar” OR “Vietnam” OR “Laos” OR “Philippines”) for 3 databases. However, for the ASEAN Citation Index, only the keyword “fall” was used due to the limited algorithm feature of the search engine (Supplementary Table 2). The keywords were inserted into electronic search engines of EBSCOHost for MEDLINE and CINAHL databases, Scopus, and ASEAN Citation Index (ACI) on 30th September 2019 and was updated up to 12th December 2020. A manual search was also conducted regarding a related review by Romli et al. (51).

### Evidence Screening and Study Selection

Two authors independently screened the titles and abstracts of studies and assessed the eligibility of the studies for inclusion against the pre-defined criteria mentioned previously. For each article, any disagreements between the 2 authors were resolved by discussion. In the first screening stage (titles plus abstracts), studies were included when any of the 2 authors agreed that they were eligible for inclusion or if there were doubts about whether to exclude them. In the second screening stage (full text), studies were included when both authors felt that they met all the inclusion criteria. A healthcare practitioner was consulted when disputed studies were identified during the full-text screening, who acted as an independent arbiter. Pre-consensus agreement on the included full-text articles between the 2 authors was calculated using percentage values. No quality evaluation was implemented as a critical appraisal of each study is not compulsory for scoping reviews (48).

### Data Extraction

The details of the studies, which included the citation, country, study objective, design, setting, instruments used, and findings, were extracted and summarized in a matrix table (46). The data extraction was done by the first author and then verified by the other authors. The findings of the review were collated into the following research categories: observational studies, interventional studies, and qualitative studies.

The stakeholder consultation (48) was conducted as part of a study reported separately (under review). The purpose of the consultation was to explore the stakeholders' perceptions of stroke rehabilitation's current practice for falls after a stroke. Concisely, the consultation implemented a qualitative study design (52). Eighteen participants from one community-based stroke rehabilitation center were purposefully selected. All participants had given informed consent before the discussions. Three focus group discussions were conducted; 2 groups combined both stroke survivors and caregivers and 1 group of only healthcare professionals. The focus group discussions were conducted following standard practice (53, 54). The sessions were conducted in a separate meeting room to ensure privacy where the participants actively engage in the discussion while two researchers acted as moderators to facilitate the sessions. Each participant was provided with a summary of the preliminary findings from the review (up to the group discussion date) and a set of open-ended questions to guide the discussions. The participants could then discuss their views in any language convenient to them as long as they understood them. The sessions were recorded using a voice recorder and through note-taking by a research assistant. The data management and analysis of this qualitative study was guided by the Sutton and Austin framework (55). The data were transcribed verbatim and coded between two authors. This qualitative study received ethical clearance from University Putra Malaysia Ethics Committee for Research Involving Humans (JKEUPM-2019-100).

### Data Analysis and Presentation of the Results

The data were narratively summarized according to pre-defined themes on fallers' characteristics, factors associated with falling, rehabilitation interventions available for falls prevention and intervention for stroke survivors, and perceptions on stroke rehabilitation and falls. The themes were developed through discussion among authors by looking into the studies' similarities and their findings. Synthesis from the review was integrated with the stakeholders' consultation. Self-reported falls and falls-related assessment tools, including their validity and reliability in the Southeast Asia context, were also documented. The interaction between themes in this scoping review was illustrated as a mapping framework.

## Results

### Study Inclusion

**Figure 1:**
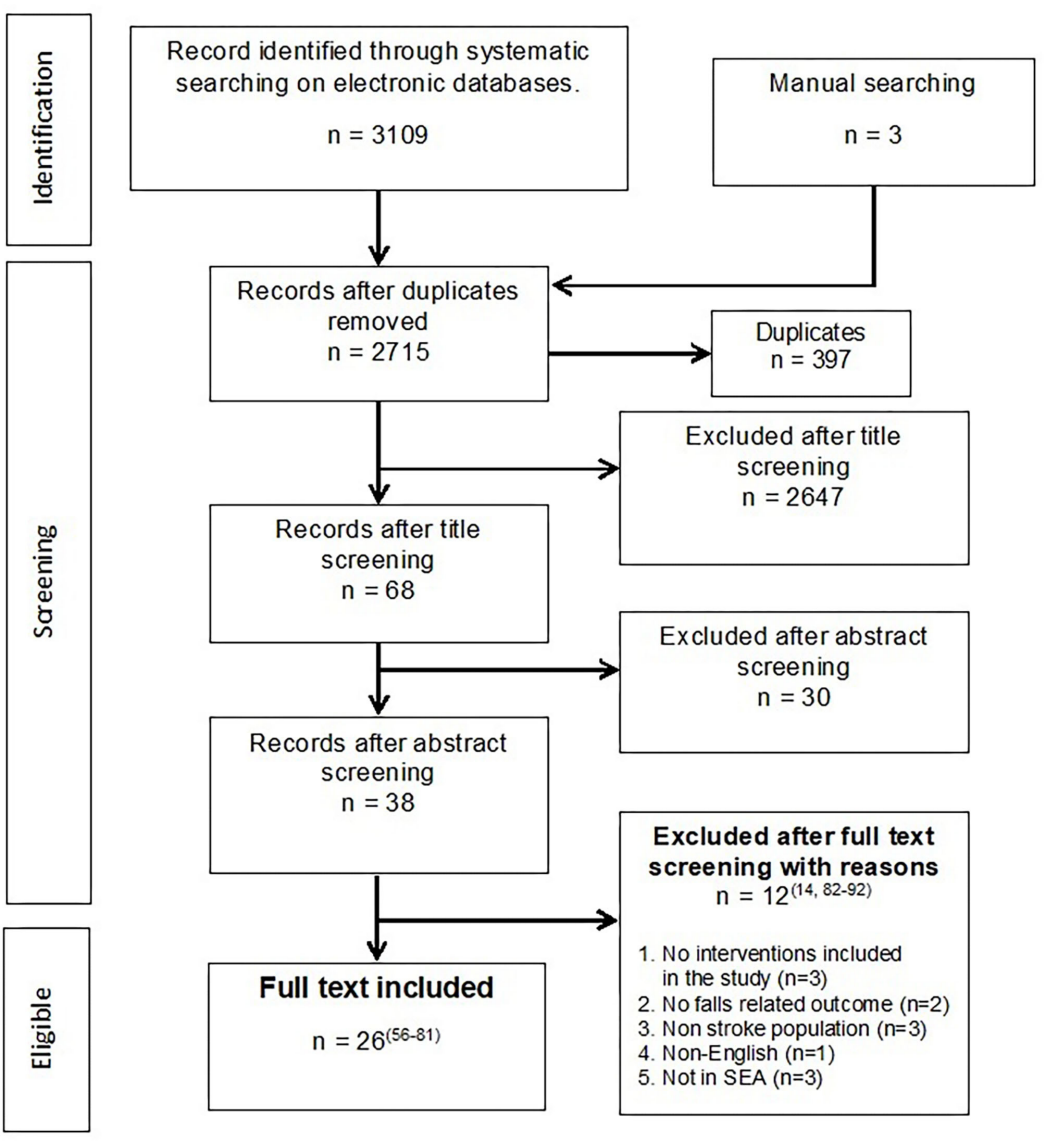
Search results and study selection and inclusion process.

The initial search yielded 3,112 unique articles, and the consensus for full-text acceptance between the two authors was 69.2% (*n* = 18/26). For disputed articles (*n* = 11), six decisions were resolved by a discussion between the 2 authors, while another 5 articles were referred to an arbiter. Of the disputed 11 articles, seven articles were accepted, and 4 were rejected. A total of 26 articles were included in the final analysis (56–81). Reasons for the exclusion of articles during the full-text screening were provided in Figure 1 (14, 82–92).

### Characteristics of Included Studies

**Figure 2:**
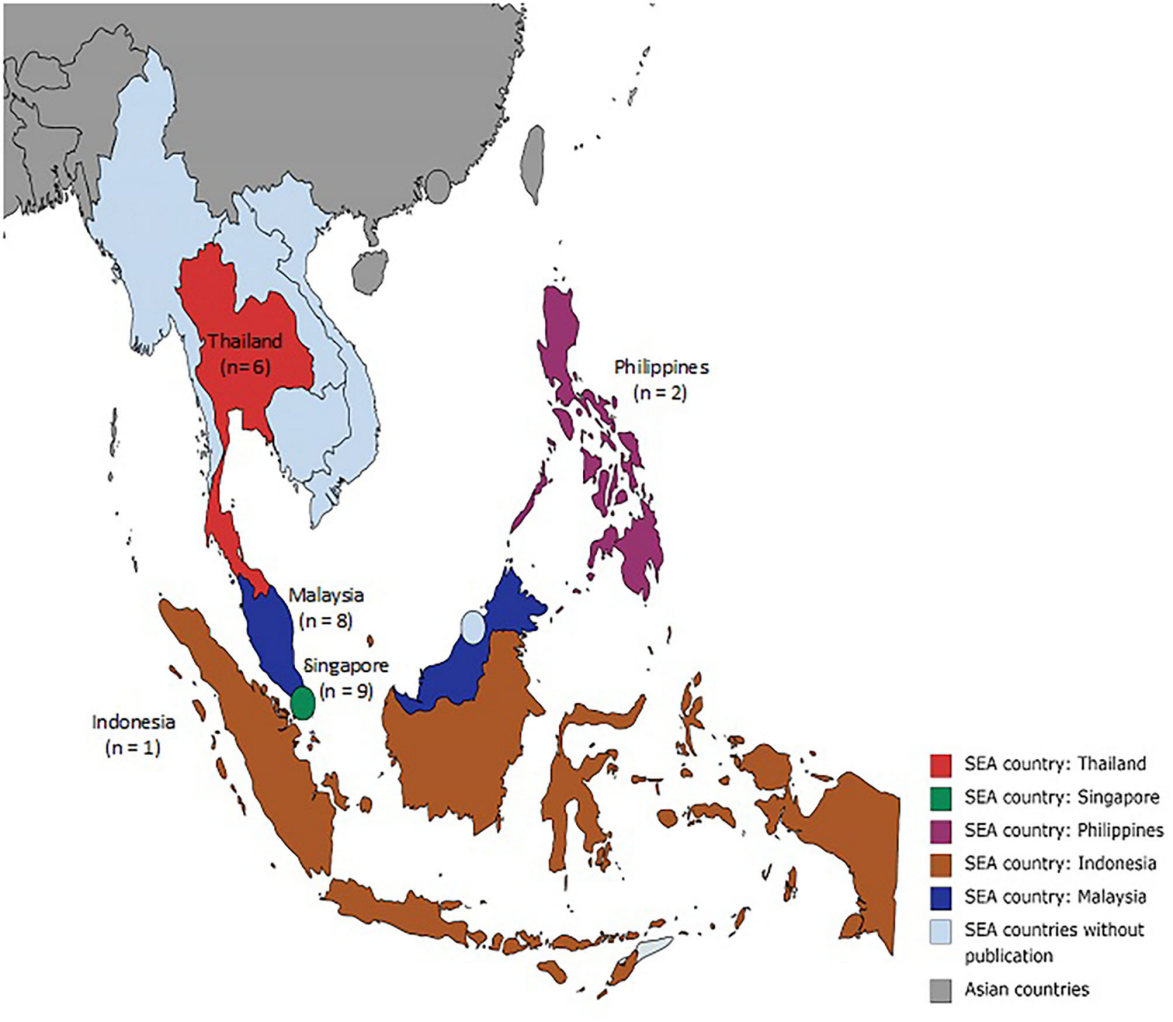
Map of Southeast Asia. Source: https://mapchart.net/asia.html.

Most of the included studies are from Singapore (63, 65, 66, 68, 71, 72, 76–78), followed by Malaysia (57, 61, 69, 70, 73–75, 80), Thailand (56, 60, 62, 64, 67, 79), the Philippines (58, 59) and Indonesia (81) (Figure 2). No articles were found from other Southeast Asia countries. The articles were published between 2006 to 2020 (Supplementary Table 3). Of the total 26 articles, five were observational studies (57, 62, 64, 66, 76), 17 were experimental (56, 58–61, 63, 65, 67, 69, 73–75, 77–81) and the remaining four were qualitative (68, 70–72). Meanwhile, 15 were clinical-based studies (56–59, 62–67, 70, 75, 78, 79, 81) and 11 studies were conducted in the community (60, 61, 68, 69, 71–74, 76, 77, 80). Most of the articles were authored by physiotherapists (*n* = 14) followed by physicians (*n* = 6), occupational therapists (*n* = 5), and an engineer (*n* = 1).

### Review Findings

Four studies (62, 64, 66, 76) reported the rate or incidence of falls. Three studies (62, 66, 76) specifically investigated the location and activities when the falls happened and four studies (62, 64, 66, 76) established the risk and associated factors related to falls. A total of 16 studies investigated the intervention for risk of falls for stroke survivors; three studies (67, 79, 81) were randomized controlled trials, two studies (56, 61) were a non-randomized controlled trial, 7 studies (60, 63, 65, 69, 75, 77, 78) were one-group pre-post experiment, four studies were case reports (58, 59, 73, 74) and one study was a protocol (80). The interventions identified from this review can be classified as conventional or technology-based methods. Conventional interventions were defined as commonly accepted interventions that are provided manually in the form of face-to-face sessions with patients, in contrast, technology-based interventions involved the use of sophisticated technology such as robots and digital facilities (i.e., virtual reality and tele-health) (93). Four studies explored the perceptions of stroke rehabilitation practices among stroke survivors, caregivers, and healthcare professionals (68, 70–72).

### Characteristics of Falls and Fallers

One study evaluated falls 6 months after stroke and found that 12.5% of the participants had fallen once, and 13.5% experienced repeated falls (64). Studies that followed up on stroke survivors over 12 months post-stroke reported that 24–28% of individuals fell at least once and 16% fell more than once (66, 76). Inpatient stroke rehabilitation reported that 15.9% had fallen during admission, 10.6% fell once, 5.3% fell twice or more, and the incidence rate was at 3.44/1,000 patient/day for all cases during the inpatient stroke rehabilitation (62). Only one study (64) evaluated fallers' characteristics: predominantly older, male, had an ischemic stroke, had left-sided hemiparesis, were using an assistive aid or wheelchair, had a greater stroke severity, had poorer cognition, and with poor lower extremity motor control. The fallers group showed less improvement in physical impairments, lower activity levels, and community participation than non-fallers (64). The fallers group also reported higher scores of falls efficacy, indicating that participants who fell had lower self-confidence and a greater fear of falling than the non-fallers (64).

### Falls Location and Activities at the Time of the Fall

Inpatient falls often happened at the bathroom (37.1%), at the bedside (22.9%), on the wheelchair (11.4%) and others (28.6%) (62). Approximately 47–86% of falls occurred at and inside of stroke survivors' homes (66, 76). Falls at home most often occurred in the toilet (35.6%), in the bedroom (16.9%), and in the living room (3.4%) (76). Thirty-two percent of falls were related to the use of stairs (66) and 20–48% occurred during walking (62, 66, 76). In addition to that, 20–22.9% of falls happened while transferring (62, 76), and the rest included activities during reaching (14.3%), dressing (11.4%), rising to stand (11.4%), showering (5.7%), turning (5.7%), and others (8.6%) (62). The most common reasons for falls as perceived by stroke survivors were loss of balance (56%) and impulsivity (15%) (76).

### Identified Risk of Falls/Associated Risk Factors for Falls

Fallers showed less improvement in physical impairments within 6 months post-stroke (64). Fallers also demonstrated significantly smaller stride length, gait speed variability, and mediolateral and vertical pelvic displacement (66). When assessing balance, fallers had significantly lower scores on the Timed-Up and Go, Step Test (66) and Berg Balance Scale (64). The risk of falling within 6 months was double when identified by balance measurements and functioning at 3 months (64). One study revealed that the transfer domain was the only significant independent factor affecting falls (76). The stroke survivors who fell were also those with more difficulties in mobility and in reintegration to everyday living (77).

### Conventional and Technology-Based Interventions

Conventional therapy consisted of either standard protocol treatments such as Motor Imagery (MI), Proprioceptive Neuromuscular Facilitation (PNF), Neuro-restoration (Bobath, Rood, Carr and Shepherd Method, and Constraint-induced Movement Therapy) or generic and conservative therapy such as exercise, functional training, strengthening exercise, task-specific gait training, and education. Most of the interventions benefited stroke survivors, and standard protocol interventions showed greater effectiveness in reducing falls, risk of falls, and fear of falling. In summary, the MI technique was found to be significant in improving gait symmetry and fall-efficacy compared to a generic physiotherapy regime (56). Similarly, PNF was assumed to benefit stroke survivors in managing the risk of falls, despite insufficient evidence (58, 59). A study in Thailand investigated Village Health Volunteers' training for stroke survivors' therapy via 1-h, weekly home visits. After 8 consecutive weeks, there was an improvement in stroke survivors' walking speed (60). One study examined multifactorial interventions for falls after stroke (77). The interventions included were exercises, peer support, homework, community mobility practice, and caregiver's education. Falls data were collected with 8 participants (four fallers and 4 non-fallers among stroke survivors) for a total of 8 months from baseline (77). At 1-month follow-up, stroke participants demonstrated positive changes in fall behavior, mobility, and goals. There were no changes among the family caregivers in terms of health status and strain except for a maid who's stress level had increased during post-evaluation, but her general health status improved during post-evaluation compared to the baseline assessment (77). Regarding the effectiveness of physiotherapy interventions on brain neuroplasticity, both the intervention and control group showed improvements in balance and functional performance. Furthermore, the neuro-restoration intervention group had greater effectiveness than the conventional physiotherapy intervention in terms of balance and functional performance but did not achieve a statistical difference in neuroplasticity regeneration (81).

Technology-based interventions identified from this review were Variable Automated Speed and Sensing Treadmill (VASST), virtual therapy exergame activity, multidirectional reach tool, home-based balance exercise, stepping response training using a Voluntary-induced Stepping Response (VSR), platform perturbation training using platform translation equipment (DST) and the BAL EX FOOT (61, 63, 65, 67, 73, 74, 78, 79). Although all interventions claimed to be beneficial to stroke survivors, there was limited evidence to support the effectiveness of technology-based interventions over conventional ones. The multidirectional reach tool, virtual therapy exergame activity, and the VASST were found to have the potential to be implemented. The use of the multidirectional tool among stroke survivors saw improvements in balance as it resulted in increased Limits of Stability (LoS) and weight-bearing squats as well as awareness and practice of behaviors that could potentially protect against falling (67). The virtual therapy exergame study reported that both the intervention and control groups had significant improvements in functional mobility and lower limb strength after the intervention phase, indicating that substituting a portion of the standard physiotherapy time with virtual reality games was equally effective in maintaining physical function outcomes and activities of daily living among community-dwelling stroke survivors (61). The VASST showed significant improvement in walking distance, gait speed, and balance of the stroke survivors (63, 65). Compliance with all 12 training sessions was 100% for all subjects, and there were no dropouts or serious adverse events when the VASST was used (65). Meanwhile, VSR and DST trainings improved protective stepping in stroke, and VSR could be a feasible alternative to equipment-based training but requires further study. The home-based balance exercise and the BAL EX FOOT cannot be recommended as these interventions had substantial methodological limitations, were still in the prototype phase, and required further investigation. In terms of feasibility, the virtual reality exergame had the most potential as it utilized commercial equipment and provided a feasible dosage similar to conventional sessions.

### Perceptions on Stroke Rehabilitation and Falls

A study by Nordin et al. (70) interviewed both rehabilitation professionals and stroke survivors, while Koh et al. (68) only focused on stroke survivors. For stroke rehabilitation in general, both professionals and survivors believed that the current stroke practice had improved over the past decade (70). However, the continuity of treatment for stroke survivors after a hospital discharge needs to improve as many survivors did not continue rehabilitation due to the lack of facilities and resources. Despite improved functional performance after inpatient rehabilitation, some patients were deterred from continuing rehabilitation as they did not feel that they gained much from their rehabilitation program. One participant commented that they did not see improvements after rehabilitation, whereby impairments could still cause a fall and result in difficulties in standing upright (68). Family-assisted therapy was one potential path to continued recovery despite the uncertainty of family members' commitment (70). Participants also claimed that family members can be overprotective and that this had discouraged stroke patients from performing home exercises, while other participants felt that the family of stroke patients had not given adequate support throughout the rehabilitation process. One stroke survivor quoted that whenever he does the exercises, his wife got angry because she was afraid that he could fall while doing the exercises. She was also worried about who will take care of the stroke survivor if he did fall while doing the exercises.

Two of the qualitative studies explicitly focused on rehabilitation for falls after stroke (71, 72). Stroke participants perceived intrinsic and extrinsic factors to be leading to falls. Muscle fatigue, decreased balance, and risky behavior (e.g., fast turning while walking) were intrinsic factors, while some of the extrinsic factors reported included environmental hazards and improper use of aids and equipment (e.g., walking aids, ankle-foot orthoses) (71). After their first post-stroke fall, almost all stroke participants showed an increased fear of falling and reported that the fall disrupted their normal daily activities. For others, it increased their dependency on caregivers and caused difficulty to conduct everyday tasks because of safety concerns (71). As part of the recovery phase, therapists who had experience in caregiver training emphasized caregivers' role in helping the stroke patients during the rehabilitation process (72). These therapists also highlighted the essential role of family members and maids in preventing falls and agreed that education and caregiver training should be further improved (72).

### Outcome Measures

The outcomes from the included studies utilized a varied number of instruments; both standardized and non-standardized. Instruments established using research internationally were considered standardized, while researcher-developed instruments developed purposely for the study or instruments used that were not cited or not provided with evidence of publication were considered non-standardized. Standardized assessments identified are listed in Supplementary Table 3, while non-standardized assessments included self-reported falls (62, 64, 66, 76–79) weight-bearing squat, gait symmetry parameters, speed and velocity stride length, step width, mediolateral and vertical pelvic displacement, external rotation of lower limb, center of pressure and limits of stability (56, 58, 59, 66, 67, 74). The list of standardized assessments utilized in the included studies is shown in Table 1, with evidence of its validation in Southeast Asia.

**Table 1:** List of Standardized Assessments.

**No**	**Domain**	**Assessment**	**Study**	**Validated in SEA**
1	Balance	Berg balance scale (BBS)	(57, 62–65, 76, 78, 81)	(94, 95)
		Time up and go (TUG) test	(61, 64, 66, 69, 79, 80)	(95–97)
		Five times sit-to-stand test (FTSST)	(58, 59, 69, 79)	(98)
		Mini/balance evaluation systems test (Mini/BESTest)	(58, 59, 79)	(99)
		Fullerton advanced balance (FAB) scale	(67)	(100)
		Thirty-Second sit to stand test (30-sSTS)	(61)	Not found
		Probalance board (Static Balance)	(61)	(101)
		Activities-Specific balance confidence (ABC) scale	(59, 79)	(102)
		Balance master	(67)	Not found
		Wii balance board (WBB)	(66)	(103, 104)
2	Walking/Gait	Ten-Meter walk test (10-mWT)	(60, 61, 63–65, 78, 80)	(98)
		Six-Minute walk test (6-mWT)	(61, 63, 65, 66, 78)	(105, 106)
		Functional ambulation categories (FAC)	(63, 65, 78)	Not found
		2-min Walk Test	(64)	(107, 108)
		Dynamic gait index (DGI)	(79)	Not found
3	Motor (general)	Fugl-Meyer assessment	(60, 64, 76, 79)	(108)
		Upright motor control test-extension (UMCT-E)	(58, 59)	Not found
		Modified ashworth scale (MAS)	(64)	Not found
		The short physical performance battery (SPPB)	(77)	(109)
4	Falls efficacy	Falls efficacy scale-international (FES-I)/Falls efficacy scale (FES-S)	(56, 64, 77)	(110, 111)
		Falls behavioral scale	(77)	Not found
5	Functioning	Modified/Barthel index	(57, 61, 62, 64, 81)	(112)
		Functional independence measure (FIM)	(76)	(113)
		Stroke impact scale subscale (participation)	(64)	(114)
		Scandinavian stroke scale	(62)	Not found
		National institute of health stroke scale (NIHSS)	(64)	(115–117)
		EuroQol-5D	(69)	(118–122)
		SF-12 health survey (SF-12)	(77)	(123)
6	Self-efficacy	Stroke self-efficacy questionnaire (SSEQ)	(80)	Not found
7	Cognitive	Mini mental state examination	(62, 64, 79)	(124–127)
8	Psychology	Geriatric depression scale (GDS)	(62)	(128–132)
		Hospital anxiety and depression scale (HADS)	(80)	(133)
9	Vision	Snellen's chart	(62)	(134)
10	Visuospatial	Line bisection test	(62)	Not found
		Copy of drawing test	(62)	Not found
		Clock drawing test	(62)	(135)
		Cancellation test	(62)	Not found
11	Pain	Visual Analog Scale	(78)	(136)
		EuroQol VAS	(69)	(137)
12	Caregiver's strain	Modified caregiver strain index (mCSI)	(77)	Not found
13	Others	Life-Space assessment	(77)	Not found
		Modified reintegration to normal living index (mRNLI)	(77)	Not found
		Goal attainment scale (GAS)	(77)	Not found

### Stakeholders' Consultation

Eleven clients and seven healthcare professionals were recruited as participants. Half of the clients were stroke survivors (*n* = 6, 54.5%), and the remaining were spousal caregivers. For healthcare-participants, the majority were physiotherapists (*n* = 5, 71.4%), followed by an occupational therapist (*n* = 1, 14.3%) and a speech therapist (*n* = 1, 14.3%). All therapists had between 1–5 years of professional experience. The findings of the qualitative study relevant to this scoping review are summarized below.

Most client-participants admitted that multiple recurrent falls occurred, consistent with the literature (66, 76). One stroke-participant said that he fell 4 times, and another caregiver-participant reported that her husband had more than 50 falls after his stroke. However, some participants did not have any falls after being discharged from the hospital. In terms of the location of falls, this review identified that most falls occurred at and inside the homes (66, 76). This is further verified by a client-participant who mentioned that he had fallen in the toilet and when going up and down the stairs in the home. Outdoor falls were also reported in a restaurant and hospital.

Stroke-participants admitted that after a fall, they became more conscious of walking freely and without assistance. One caregiver whose husband was a faller said that falling had made him even more afraid to walk. Another stroke survivor also echoed that his number one concern after a stroke was the risk of falls after having several near misses. Furthermore, the caregivers also emphasized that they did not let their spouses do daily activities alone to avoid unwanted falls. This supports the study by Jalayondeja et al. (64) in which fallers participated less in activities and the community, had lower self-confidence and a greater fear of falling. The participants also recognized that physical impairments play a role in falls, as stated in the literature (64, 66). However, there were additional factors not identified in the literature but had a significant impact, such as the environment (e.g., uneven road), communication, and cognition. One caregiver mentioned that her husband's memory was not good, and he had difficulty distinguishing between right and left. According to the caregiver, this could have contributed to falls at home.

In terms of interventions received, all participants agreed that most of the interventions listed in the review were conducted except the technology-based interventions. However, the list was not exhaustive. Many other activities (i.e., domestic and leisure activities) were also implemented. This is because the interventions implemented in real practice targeted holistic objectives rather than only focusing on falls. This was mentioned by one healthcare-participant where preventing falls came indirectly when an improvement in other aspects was achieved. This was also agreed by client-participants where they viewed that improving their social life was more important than preventing falls after stroke. Environment and home hazards were somehow overlooked, both from gathered literature as well as by the participants. Shortage of manpower, cost, and time constraints to conduct home assessments were some of the reasons stated by healthcare-participants for the less attention to home hazards. For client-participants they perceived that home visits and modifications were not necessary, were expensive, and they did not appreciate the undesired aesthetic value (i.e., hospital-like environment) of their homes after the modifications.

### Interaction of Information in the Scoping Review

**Figure 3:**
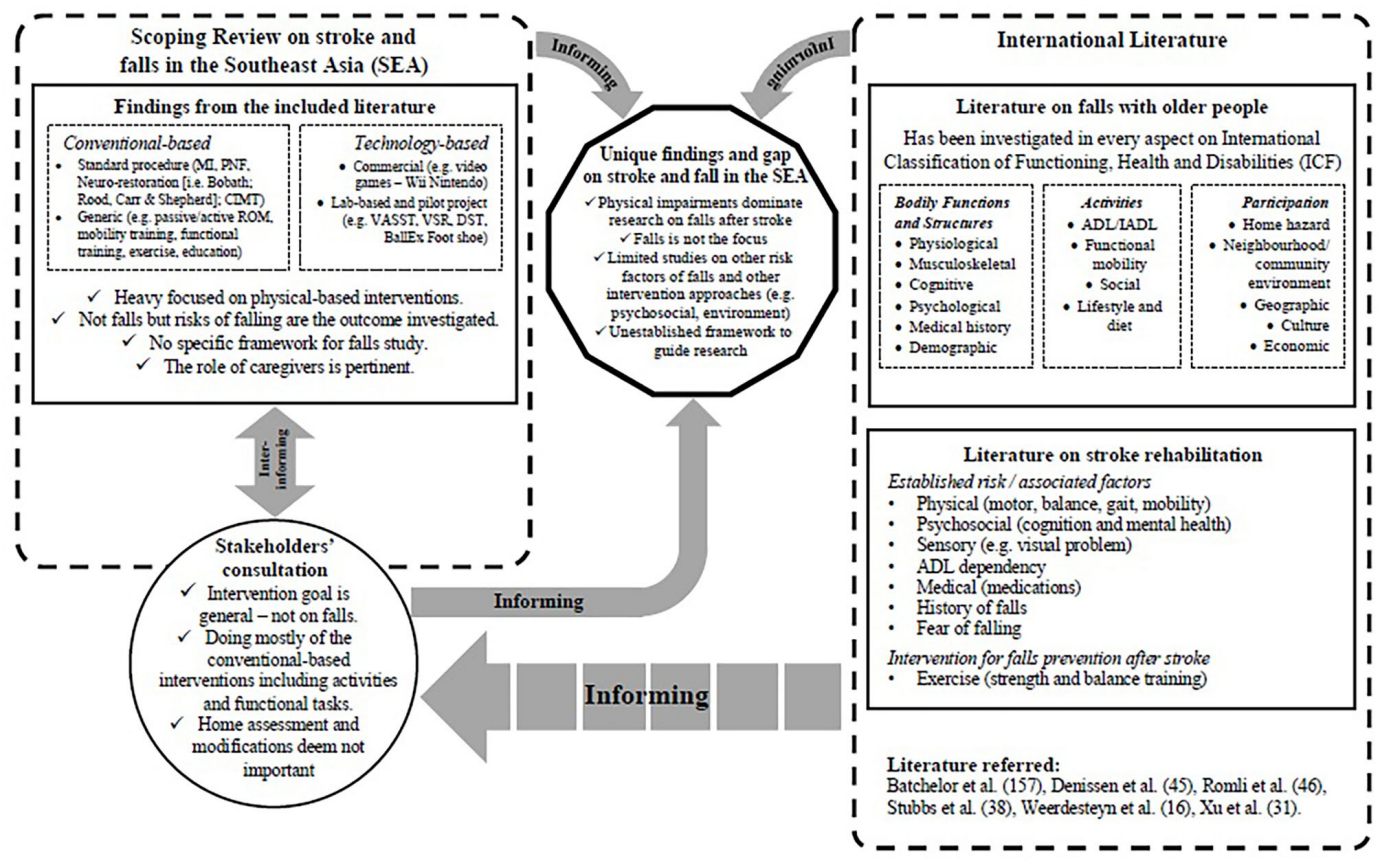
Mapping of information of the scoping review.

The information gathered within this scoping review is mapped to show the linkage, as illustrated in Figure 3. The figure presents the interaction of the combined information from the review findings, consultation outcome, and the comparison of stroke and falls literature within the international context.

## Discussion

This scoping review addresses a new research area related to falls and falls risk with stroke survivors in Southeast Asia. This scoping review also provides a comprehensive understanding of this topic and identified critical gaps (48). Consultation with the stakeholders provided an added value (48, 138). The scoping review was comprehensive but inadequate to gain a realistic view of what occurs in practice in Southeast Asia compared to the feedback from the consultation group (138). Compared with international literature pertaining to falls and stroke, research in Southeast Asia was one dimensional, not comprehensive, and has yet to mature (30, 31, 45, 139). One of the reasons for this is that falls are still given less priority either in stroke rehabilitation research or practice. Hence, falls prevention is perceived as a secondary outcome for stroke rehabilitation as the other interventions received greater attention. This scoping review indicates how falls are set aside in stroke rehabilitation practice. Although stroke rehabilitation targets physical and functional improvement in general, it fails to benefit falls prevention and intervention specifically. Furthermore, a limited number of health professionals are involved in fall prevention studies, which results in the limited exploration of potential interventions and fewer research initiatives (140, 141). Conducting low-quality research isolated from the real-world limits the application of the findings (142, 143). Therefore, awareness about falls needs to be elevated, and interdisciplinary research should be encouraged.

### Features of Stroke and Falls Research in Southeast Asia

This study only found studies from 5 countries in Southeast Asia that are actively investigating stroke and falls. This trend was similar to the other reviews in Southeast Asia on general stroke research (144) and falls in older people (46). Publications in high-impact journals from Singapore, Thailand, and Malaysia may result from these countries having higher incomes and a higher percentage of Gross Domestic Product (GDP) being allocated to research and development (144). Limited funding hinders the initiative for distinctive efforts of stroke rehabilitation for falls prevention to be explored truly. This could cause literature bias as references on stroke rehabilitation only come from higher-income countries and might not be transferable, relevant, or appropriate for other Southeast Asia countries.

More than half of the studies were conducted in a clinical setting. This could be due to the limited rehabilitation services in the community, thus making it difficult to conduct programs and research (70). However, over the past decade, the number of community centers dedicated to stroke has increased to meet the need for a growing number of stroke survivors (69). Despite its growing significance, minimal empirical evidence on the benefits of these initiatives is available to date. Most community services are currently administered by non-governmental organizations and only used later or in chronic stage post-stroke. As Community-Based Rehabilitation (CBR) is a continuity of care after discharge from the hospital, it is essential to have healthcare professionals working in a CBR. Advanced training is warranted for healthcare professionals including falls prevention and intervention, community reintegration and participation. These aspects are most important for stroke survivors transitioning from the clinical setting to the community.

Falls rates and incidences reported in this study were lower when compared with international literature in which they reported that 25–37% of stroke survivors fell during the first 6 months after stroke and 40–50% fell between 6 and 12 months, respectively (27, 28, 145, 146). This is consistent with other Asian findings where fall prevalence is often lower than those reported in other international studies (46, 147). The low number of falls could be due to the dependency associated with Asian culture, whereby ill patients are cared for by their family members, particularly their spouse or children. Besides this, the fear of falling among stroke survivors and caregivers could also explain the low numbers of falls reported. Stroke survivors tend to limit themselves from walking and carrying out daily activities as these activities are perceived to be dangerous and may compromise their balance and lead to falls. While these routines are perceived to be appropriate to prevent falls, however, the restriction of activities caused by the fear of falling could eventually lead to increased dependency and anxiety, and decreased functional mobility, ADL, and community participation (148). In the long run, stroke survivors will be trapped into disability-worsened conditions and consequently increase caregivers' burden.

### Falls Prevention Is a Secondary Outcome in Stroke Rehabilitation

The seriousness of falls is neglected when considered alongside the numerous stroke impairments such as physical impairments and functions, problems in cognition, and daily activities. This could be related to the observability and tangibility of impairments due to a stroke, which provides constant attention compared to falls. Falls are usually denied and considered an inevitable event and only a by-product of impairments due to aging (30, 149–151). However, falls are an underlying sign of a greater problem and warrant prompt attention (30).

Stroke survivors and caregivers stated that the rehabilitation they undergo most often is to improve physical impairments and increase independence. Furthermore, activities and interventions are conducted to increase strength, balance, and gait speed, all of which are risk factors for falls. However, stroke rehabilitation, in general, has not been successful in precise target falls prevention. Indeed, although most stroke rehabilitation programs may indirectly target fall prevention, the goal is commonly implemented as a subcomponent rather than as an intervention of its own. This leads to falls prevention becoming a secondary outcome for stroke rehabilitation. Hence, it is crucial to make falls prevention a primary goal in stroke rehabilitation.

### An Over-emphasis on Physical Rehabilitation for Falls Prevention

Stroke survivors and caregivers expressed their views regarding undergoing rehabilitation to prevent falls. Healthcare professionals also agreed that rehabilitation for stroke survivors is often implemented according to the client's needs. The effectiveness of rehabilitation methods on physical, psychosocial, and cognitive outcomes varies according to the numerous rehabilitation methods used (152). However, this perspective is not comprehensive for falls prevention and intervention.

Most interventions implemented in this review focused on improving the physical impairments of stroke survivors. This is unsurprising as research on falls and stroke is still heavily focused on intervening for physical impairments (30). Exercise was found to be effective in improving balance and reducing spasticity, while other techniques such as functional training, task-specific gait training, and education assisted in the overall physical performance of the stroke survivors. Technology-based interventions also focused on physical rehabilitation, especially for lower body functions. While technology is considered supplementary modalities for therapy, technology should be explored beyond physical training (153). There were relatively fewer studies on psychosocial, environmental, and cognitive factors in this review. Even literature on stroke rehabilitation, in general, emphasized the physical motor aspects of intervention, with only some focusing on cognitive factors (154).

The majority of the studies were authored by physiotherapists. This could explain why physical impairment aspects still dominate stroke and falls research in this region. The risk and associated factors of falls in this review also illustrated similar findings as with many previous studies (21, 155), where physical impairments were the main risk factors for falls after stroke. This over-emphasis on physical rehabilitation is not only exclusive to stroke rehabilitation but also appears in other areas such as in cancer rehabilitation (152). This is consistent with Loh and Musa (152) who examined rehabilitation for cancer patients and reported that studies investigating physical outcomes dominate the literature. A narrow view of the role of rehabilitation and the heavy influence of the medical model may contribute to this standpoint. The medical model views clients as a specific problem of disease and disability and aims more toward curing the disease. This leads to the segregation of the expertise of health professionals (156). However, several cohort and case-control studies have established other risk factors for falls, which include fear of falling (157, 158), depressive symptoms (159), a fall history during hospitalization (20, 160), motor and sensory impairment (157), and environmental hazards (161). This necessitates research on stroke rehabilitation and falls to be open to other disciplines, and interdisciplinary research should be encouraged.

Compared to research on stroke, studies on falls with older people have received greater interest from researchers of various disciplines such as social sciences, human ecology, economics, built environment, engineering, and health sciences other than medical (162). As a result, this has extended the range of factors contributing to falls and should be modeled for stroke and falls research. Currently, the study of falls and stroke remains predominantly investigated only among the medical disciplines. Overall, the lack of evidence for non-physical rehabilitation methods highlights the lack of research work that extends beyond the rehabilitation methods for physical after-effects (152). Contemporary views of health have now shifted toward the biopsychosocial model of illness which views clients as holistic beings encompassing the need to treat their disability and empower them to function in society (163).

### Under-recognizing the Importance of Environmental Factors as a Risk Factor for Falls

Current evidence shows other established risk factors for falls and stroke such as fear of falling, depressive symptoms, history of falls during hospitalization, and having cognitive, motor, and sensory impairment (20, 31, 158, 159). However, home hazards are significantly absent as presently there are no studies available that investigate this factor in stroke and falls research. Conversely, there is evidence about the role of home hazards in fall management, and standardized instruments are also available to measure and establish the relationship between home hazards and falls (164–166). No studies from this review were found to have assessed environmental factors as one of the risk factors for falls. In addition, reducing home hazards has received skeptical reviews of its effectiveness in preventing falls, although the impact is apparent. Participants also reported many issues regarding home assessment and modifications after stroke. These issues include a lack of understanding of the concept and implementation of home assessments and modifications and limited health personnel to conduct the assessment and intervention.

Another reason for the under-recognition of the importance of environmental factors is that there are not many home assessments available for use in Southeast Asia. Most assessment tools derived from Western countries and need to be adapted and validated before use. However, limited resources can hinder the process of adapting and validating these assessment tools (46, 166, 167). Although there is an increasing number of stroke survivors, limited staff and resources (70) signify the current impracticality to conduct home visits for all stroke survivors living in the community. Thus, there is a need to select and validate a high-quality self-reporting screening tool to assess home hazards that can be utilized for the stroke population and their caregivers. The use of home hazards instruments sensitive to cultural settings may capture more valuable and meaningful hazards (168, 169). With home hazards, healthcare professionals should focus on clutter, assistive devices (i.e., mobility aids, shoe), and the functional aspects of the person-environment fit, and in particular, attention should be given to the bathroom and stairs area as these were also found to be related to falls (170, 171). The use of technology such as photographs and videos were found to be the most credible and cost-saving method to conduct a home assessment while substituting a physical home visit (166). Therefore, this may improve the constraints of home hazards evaluation practices.

### Establishing a Comprehensive Framework to Guide Stroke and Falls Research

A strong underlying framework should become a foundation for Southeast Asian researchers. From the rehabilitation perspective, patients' functioning and health are associated with, but not merely a consequence of, a condition or disease. Furthermore, functioning and health are seen in association with a condition, personal and environmental factors and the rehabilitation context (172). The International Classification of Functioning, Health and Disabilities (ICF) document is a good guideline for steering fall research (173). The ICF is a comprehensive document that views health and disabilities on three main aspects: (i) body function and structure, (ii) activities, and (iii) participation, where these three aspects are influenced by personal and environmental factors (174). As falls risk is multidimensional, the ICF is suitable to investigate falls in the clinical practice thoroughly (175).

A study by Cieza et al. (175) demonstrated that falls among older women were associated with a wide range of health, functional and environmental factors consistent with the ICF classification. The study also established that while the ICF was developed as a classification system, it remains a useful framework to use as a guide for future studies related to falls risk in a community population. However, the lack of detail within the ICF about personal factors was an issue (175) because critical personal factors that determine increased falls risk may extend beyond the ICF classification factors and may include unique, individual features associated with a person-centered practice (176).

This scoping review revealed that all of the articles included were authored by a variety of professions; however, each article was dominated by only one single discipline. Involvement of other disciplines within and outside of healthcare (e.g., built environment, psychology, architecture, engineering, and social care) is warranted to study falls and stroke. Multidisciplinary and interdisciplinary research should be encouraged. Certainly, interdisciplinary research opens new paradigms and develops more creative and innovative solutions for a particular problem, including falls prevention and intervention (37, 38, 177).

Studies on falls with older people show the benefits of the involvement of various disciplines in falls prevention and intervention. It has also been found that observing the local and cultural context to identify novel factors will strengthen the knowledge and benefits the clients and stakeholders (46, 162, 178). Healthcare practices that are culturally sensitive were found to improve awareness of falls, clients' satisfaction, and other patient outcomes (178–181). Therefore, it is hoped that this scoping review provides an insight for future research to consider both elements when conducting studies on falls and stroke.

### Outcome Measures

This review identified that many of the outcome measures used in the studies were validated. The validation process included translation into the respective languages and cultural adaptations. However, only a few countries in the Southeast Asian region have conducted validation studies on these instruments. Moreover, many validated measures were conducted with the general population rather than with specific groups and settings.

These findings are similar to a review by Romli et al. (46) in which assessments used in their review were published standardized tools, but many were invalidated for Southeast Asia. This could be due to cost, practicality, clinical relevance, and a lack of knowledge over which outcome measures to choose (182). Furthermore, the use of non-standardized or generic tools may not provide accurate assessment information and may lead to under or overestimation of falls risk (165, 166, 176). Future research in Southeast Asia should cover every domain in the ICF as this has been proven to facilitate fall research (172, 174). Thus, this requires an assessment tool, especially for functional performance and participation, to be utilized. A comprehensive list of functional performance assessments has been made available (183). Researchers should use standardized assessments where possible and thoroughly investigate such tools' validity and reliability in the local context (164).

### Limitation and Recommendation

The scoping review is also characterized by certain limitations. The main limitation of this review is that many of the studies did not report on falls but focused more on the risk and associated factors of falls. Thus, the results are inconclusive in examining the effectiveness of stroke rehabilitation for reducing falls. Furthermore, only publications in English were included in the database. Although the majority of studies in this field are currently published in English, this could mean that some studies from individual Southeast Asia countries have not been included in the database. Apart from that, since the nature of the review appears to include a summary of the evidence from a limited range of publications, a comprehensive evaluation of the studies' validity and possible bias evaluation should be carried out.

## Conclusion

This review describes stroke rehabilitation's current scenario for falls and the risk of falls in Southeast Asia. Despite falls in Southeast Asia being a significant issue, the management of falls in stroke has not been comprehensively investigated. Research that specifically targets falls prevention among the stroke population in Southeast Asia is warranted to increase the awareness of the importance of fall prevention in stroke. The stakeholders, including the stroke survivors, caregivers, and health practitioners, perceived falls as a serious event, however, their actions are not aligned with that perception. Furthermore, falls prevention strategies are only considered secondary outcomes and indirect goals achieved from rehabilitation such as improved balance from functional activities training. The over-emphasis on physical rehabilitation for falls after stroke necessitate the exploration and investigation of a broader spectrum of risk factors that extends holistically in the aspects of psychosocial, cognitive, and the environment. Attention to established risk factors and characteristics of falls derived from studies among the older population could also aid in implementing effective fall management programs for stroke survivors as it is evident that falls are multidimensional in nature. A more comprehensive review on stroke rehabilitation in general in Southeast Asia should be conducted to map out the similarities and differences of practices in place, providing novel insights for fall prevention strategies.

## Data Availability Statement

The raw data supporting the conclusions of this article will be made available by the authors, without undue reservation.

## Author Contributions

MR directed the development of the inclusion/exclusion criteria and search strategy. HA implemented the search in the databases and lead the writing of the methods section of the manuscript. HA and MR collaborated on data analysis and drafting of the manuscript. TH, MS, and LM provided input on the direction of the data analysis and revised continuous amendments of the manuscript draft. All authors read and approved the final manuscript.

## Conflict of Interest

The authors declare that the research was conducted in the absence of any commercial or financial relationships that could be construed as a potential conflict of interest.
