# Supplementary material for: Stroke Rehabilitation for Falls and Risk of Falls in Southeast Asia: A Scoping Review With Stakeholders' Consultation
Source: Front Public Health. 2021 Mar 3;9:611793. doi: 10.3389/fpubh.2021.611793 (PMC7965966; doi:10.3389/fpubh.2021.611793)
Supplement: Supplementary file 2 [file Table_2.docx]

**Supplementary Table 2-Search Terms**

| (Search Engine) Database | Search Terms |
| --- | --- |
| *(Scopus)*  Scopus | (TITLE-ABS-KEY ("cerebrovascular accident" OR "CVA" OR "stroke" ) AND ("fall*" OR "fall* prevention" OR "fall* intervention" OR "accidental fall*" OR "risk of fall*" OR "fall* predictor*" OR "prevalence of fall*") AND (“rehabilitation” OR “occupational therap*” OR “physiotherapy*” OR “physical therap*” OR “speech language patholog*” OR “speech therap*”) AND (“Southeast Asia” OR “Malaysia” OR “Singapore” OR “Thailand” OR “Brunei” OR “Indonesia” OR “East Timor” OR “Cambodia” OR “Myanmar” OR “Vietnam” OR “Laos” OR “Philippines”)) |
| *(ASEAN Citation Index)*  ASEAN Citation Index | “fall” |

| (Search Engine) Database |  | Search Terms |
| --- | --- | --- |
| *(EBSCOHost)*  MEDLINE  CINAHL | 1 | "cerebrovascular accident" |
|  | 2 | "CVA" |
|  | 3 | "stroke" |
|  | 4 | #1 OR #2 OR #3 |
|  | 5 | "fall*" |
|  | 6 | "fall* prevention" |
|  | 7 | "fall* intervention" |
|  | 8 | "accidental fall*" |
|  | 9 | "risk of fall*" |
|  | 10 | "fall* predictor*" |
|  | 11 | "prevalence of fall*" |
|  | 12 | #5 OR #6 OR #7 OR #8 OR #9 OR #10 OR #11 |
|  | 13 | “rehabilitation” |
|  | 14 | “occupational therap*” |
|  | 15 | “physiotherapy*” |
|  | 16 | “physical therap*” |
|  | 17 | “speech language patholog*” |
|  | 18 | “speech therap*” |
|  | 19 | #13 OR #14 OR #15 OR #16 OR #17 OR #18 |
|  | 20 | “Southeast Asia” |
|  | 21 | “Malaysia” |
|  | 22 | “Singapore” |
|  | 23 | “Thailand” |
|  | 24 | “Brunei” |
|  | 25 | “Indonesia” |
|  | 26 | “East Timor” |
|  | 27 | “Cambodia” |
|  | 28 | “Myanmar” |
|  | 29 | “Vietnam” |
|  | 30 | “Laos” |
|  | 31 | “Philippines” |
|  | 32 | #20 OR #21 OR #22 OR #23 OR #24 OR #25 OR #26 OR #27 OR #28 OR #29 OR #30 OR #31 |
|  | 33 | #4 AND #12 AND #19 AND #32 |
