# Supplementary material for: Stroke Rehabilitation for Falls and Risk of Falls in Southeast Asia: A Scoping Review With Stakeholders' Consultation
Source: Front Public Health. 2021 Mar 3;9:611793. doi: 10.3389/fpubh.2021.611793 (PMC7965966; doi:10.3389/fpubh.2021.611793)
Supplement: Supplementary file 3 [file Table_3.docx]

**Supplementary Table 3- Summary of Studies**

| Study Protocol | | | | | | | | |
| --- | --- | --- | --- | --- | --- | --- | --- | --- |
| Author | Country | Study Objective | Study Design | Setting and Participants | Instruments | Intervention | | Findings |
| Kei et al. (2020)  (80) | Malaysia | To assess the effectiveness of  post-discharge Home-based Therapy (HBT) in comparison to usual practice on functional outcome (mobility and gait speed), self-efficacy and anxiety level  among stroke survivors. | Randomized Control Trial (RCT) | Sample: 42 stroke survivors  Setting: Universiti  Kebangsaan Malaysia Medical Center and Cheras Rehabilitation  Hospital | Time up and go (TUG) test, 10-meter walk test (10mWT), Stroke Self-Efficacy Questionnaire (SSEQ), Hospital Anxiety and Depression Scale  (HADS) | Duration: 45 to 60 minutes, 3 times a week for 12 weeks.  Intervention group: 9 strengthening, walking, task-related and balance exercises.  Control group:  Exercises as prescribed by the therapist prior to discharged from  physiotherapy service. | | - |
| Interventional Studies |  |  |  |  |  |  | |  |
| Author | Country | Study Objective | Study Design | Setting and Participants | Instruments | Intervention | | Findings |
| Rahayu et al. (2020) (81) | Indonesia | To investigate the effects of physiotherapy interventions on brain neuroplasticity by  evaluating the brain plasticity regeneration, balance, and functional ability. | RCT | Sample: 64 stroke survivors. Age: Intervention group (58.84 ±8.68 years), control group (59.93 ±10.64 years)  Setting: three hospitals in Surakarta region | Berg Balance (BBS), Barthel Index (BI) | Intervention group: Neuro-restoration intervention which consists of Bobath, PNF, Rood, Carr and Shepherd and CIMT.  Control group: conventional physiotherapy  Duration: 7 days | | Intervention group: improvements in functional performance (P=0.008) and balance (P =0.016), but not in  neuroplasticity regeneration  (P=0.07) |
| Chayasit et al. (2020) (79) | Thailand | To compare the immediate effects of voluntary-induced stepping response training (VSR) and  DynSTABLE perturbation training (DST) on protective stepping in patients with stroke. | RCT | Sample: 34  chronic stroke patients Age: VSR group (66.5 ±10.3 years), DST (68 ±10.9 years)  Setting: a neurorehabilitation  center | Mini Mental State Examination (MMSE),  Activities-specific Balance Confidence (ABC), Fugl-Meyer (FMA-LE), Five Times Sit-to-Stand Test (FTSST), items 16–18 of the Balance Evaluation Systems Test (BESTest), TUG test and Dynamic Gait Index (DGI), Step length, width, number of steps and Center of Mass (CoM), Self-Reported fall history within the past 12 months and the fear of falling was identified by asking the question ‘are you afraid of falling?’ | Intervention: Voluntary-induced stepping response (VSR) and DynSTABLE perturbation training (DST). Each participant was given a total of 60 minutes of training which included:  a. warm up (7 minutes)  b. VSR/DST (3 sessions, a total of 50 minutes)  c. cool down (3 minutes) | | Both groups: wider step width (P<0.05, 95% CI 1.46 to 4.56, Cohen’s d=0.31) and longer step length of affected leg when stepping (P<0.01, 95% CI: 2.4 to 10.23, Cohen’s d=0.51).  DST group: significant interaction effect (P<0.01), longer overall step length (P<0.001, 95% CI: 3.12 - 7.87, Cohen’s d=0.54), significance between time and training group (P<0.02) indicated improved CoM of stepping limb’s heel of mostly unaffected leg (P<0.01, 95% CI, 13.94 to 48.79, Cohen’s d=0.48). |
| Khumsapsiri et al. (2018) (67) | Thailand | To investigate the effect of training using a new multidirectional reach tool on balance in individuals with stroke. | RCT | Sample: 16 stroke patients  Age: Experimental group (61 ±10.59 years), Control Group (57.50 ±9.79 years)  Setting: a university's physical therapy clinic, hospital rehabilitation unit. | Balance Master, Weight‐bearing squat, Fullerton Advanced Balance scale (FAB) | Intervention Group: Multidirectional reach training (30 minutes) and conventional physical therapy (30 minutes)  Duration: 3 days a week for 4 weeks  Control Group: Conventional physical therapy  Duration: 30 minutes per day, 3 days a week for  4 weeks. | | Intervention group: a. endpoint excursions at backward, mean difference (95% CI) = 0.36 (0.70 to 0.03), P=0.034  b. less affected side, mean difference (95% CI) = 0.10 (0.19 to 0.01), (P= 0.032)  c. maximum excursion on the less affected side, mean difference (95% CI) = 0.21 (0.34 to 0.08), (P=0.003).  d. weight‐bearing squat of affected side at 0° mean difference (95% CI) = 0.10 (0.19 to 0.01), (P=0.034), 30° mean difference (95% CI) = 0.12 (0.24 to 0.10) (P=0.034), and 90°mean difference (95% CI) = 0.15 (0.25 to 0.06) (P=0.003) |
| Pheung-Phrarattanatrai et al. (2015) (56) | Thailand | To investigate effect of gait training with motor imagery (MI) on gait symmetry and self-efficacy of falling in stroke patients. | NRCT | Sample: 14 stroke patients  Age Median (Q1-Q3):  MI group (60, 51-63 years), Control Group (60, 53-63 years),  Setting: Neurological Clinic, Physical Therapy Center, Mahidol University | Gait symmetry parameters (step length, step time, stance time, and vertical impulse symmetries) and Falls Efficacy Scale- International (FES-I) | Intervention Group: Strengthening exercise,  functional weight bearing exercise, task specific gait training and MI training.  Control Group: Strengthening exercise,  functional weight bearing exercise, task specific gait training.  Duration: 50 minutes for both groups. An additional 20 minutes of MI training for the intervention group.  Outcome was measured three times (pre-, intermediate- (2nd week), and post-trainings.  (4th week)). | | Within MI group:  a. step length symmetry (P=0.018) at week 4 when compared with week 2.  b. FES-I at post-training when compared with pre- (P=0.017) and intermediate trainings (P= 0.017).  Both groups: significant difference of the FES-I at post-training (P=0.047). |
| Singh et al. (2013) (61) | Malaysia | To determine changes in physical function and ADL when substituting a portion of the standard physiotherapy time with virtual reality games | NRCT | Sample: 28 stroke survivors  Age: Experimental  Group (65.4 ±9.8 years), Control group (67.0 ±8.4 years)  Setting: community stroke rehabilitation  centers | TUG, Thirty-second Sit to Stand Test (30sSTS), 10mWT, 6mWT, Static Balance Ability Using Pro-Balance Board and BI | Experimental Group: 30 minutes of Virtual Reality (VR) balance games, in addition to 90 minutes of standard group exercise therapy.  Control Group: routine standard group exercise therapy: self-stretching and strengthening exercises, coordination and balance exercises, functional exercises, and endurance training.  Duration: 12 therapy sessions: 2-hour sessions, twice per week for 6 continuous weeks. | | Within-subject effect: TUG; F (1, 26) = 5.83, P=0.02; and 30sSTS; F (1, 26) = 13.50, P=0.001. |
| Chua et al. (2020) (78) | Singapore | To study the  feasibility and safety of the VASST II using a physiotherapist supervised training protocol in an outpatient rehabilitation clinic and to determine the VASST II’s preliminary efficacy in subacute  and chronic stroke patients with a predominant hemiplegic  pattern of weakness. | Pre and Post | Sample: 11 sub-acute and chronic stroke survivors  Age (median): 53.0 (IQR: 22 years)  Setting:  Tertiary rehabilitation center | 6mWT, 10mWT, Functional Ambulation Categories (FAC), BBS, Visual Analog Scale (VAS) and self- reported falls. | Duration: physiotherapist-supervised training on VASST II for 60 minutes daily, 3 times  per week, for 5 weeks (a total of 15 hours).  Outcome was measured at Week 3, Week 6, Week 12, and Week 24. | Week 6 (from baseline): significant (mean ±SD) improvements in the 6mWT (44.9 ± 49.2m; P = 0.003), 10mWT (0.12 ± 0.15 m/s; P = 0.016), and BBS score (42 ± 10; P = 0.003).  Week 12 (from baseline): a mean ±SD improvement in 6mWT (56.2 ±63.8m; P= 0.007), 10mWT (0.17 ± 0.21 m/s; P= 0.018) and in BBS score (5 ±3 points; P = 0.005).  Week 24 (from baseline): An increase in the 6mWT (mean ± SD of 58.8 ± 80.4m; P = 0.013) and BBS (mean ± SD of 2 ± 2 points; P = 0.012). | |
| Xu et al. (2020)  (77) | Singapore | The acceptability and feasibility  study carried out to assess whether (a) the Stepping On after Stroke (SOAS) program  meets the health, social, and care needs of  community-dwelling stroke survivors and also their caregivers  (b) the outcome measures are relevant  and applicable; and (c) the program is culturally.  relevant within the Singapore context. | Pre and Post | Sample: 8 stroke clients, 6 family caregivers  and 1 maid from two groups  Age: 65 ±10.5 years  Setting:  community rehabilitation  centers, one stroke support center, and a community  hospital | Self-reported monthly falls calendar, FES-I, Falls Behavioral (FAB) Scale, Life-Space Assessment (LSA), modified Reintegration to Normal Living Index  (mRNLI), Goal Attainment Scale (GAS), The Short Physical Performance Battery (SPPB), SF-12 Health Survey  (SF-12) and the modified Caregiver Strain Index  (mCSI) | Duration: 7 weekly group sessions focusing on different falls-prevention topics delivered by experts from different fields and facilitated by a trained program leader.  These were followed by a home visit 4 weeks post-intervention, a booster session 3 months post-intervention, and a follow-up phone call 6 months post-intervention.  The family caregivers and/or maids were invited to the weekly sessions. In addition, caregivers were invited to participate in two education sessions in week 1 and week 7 of the intervention. | Four (50%) recurrent fallers and four (50%) non-fallers.  At 1-month follow-up: stroke participants demonstrated positive changes with large effect sizes in the FAB Scale (P=0.041; d= 0.92), LSA (P=0.028; d=0.83) and GAS (P=0.012; d=2.58) when  compared to the baseline. There were no differences at 1-month follow-up for FES-I, mRNLI, and SPPB, and no changes among the family caregivers in both the SF-12 (P=0.833) and mCSI  (P=0.786). | |
| Nordin et al. (2019) (75) | Malaysia | To assess the feasibility and usefulness of a low-frequency group  exercise to determine its suitability as an approach to facilitate exercise engagement among stroke survivors. | Pre and Post | Sample: 41 stroke survivors  Age: 59.34 ±10.02 years  Setting: an outpatient physiotherapy unit at a university hospital | BBS, FTSST and 10mWT | Group exercise  Duration: once a week, 90 minutes per session, 12 weeks. | Increased BBS score from 51.00 (24–56) to 54.00 (34–56) (Z = -3.88, P <0.001), FTSST score reduced by 3.40 s (Z = -4.69, P <0.001), and the walking speed improved from 53.09 (12.6–120) to 61.22 (13.6–107.14) m/min (Z = -3.25, P = 0.001) after 12 weeks of exercise. | |
| Chinchai et al. (2017) (60) | Thailand | To investigate the effect that rehabilitation education provided to village health volunteers would have on walking speed and upper extremity function in stroke survivors who stay at home | Pre and Post | Sample: 27 stroke survivors  Age: 31 years and above  Settings: Community | 10mWT, Fugl-Meyer | a. Rehabilitation education Duration: 7 hours in 1 day. The education consists of basic knowledge of stroke, gait training and UE function training.  b. Home Visits  Duration: Once a week (1 hour per visit), 8 consecutive weeks. | The participants used significantly less time for walking at post-test than at pre-test (P <0.05). Gait speed at pre-test was 0.17 meters per second (m/s) and increased to 0.19 m/s at post-test. | |
| Nordin et al. (2016) (69) | Malaysia | To evaluate the outcome of CBR on physical function and health status among chronic stroke survivors who lived at home post hospital discharge. | Pre and Post | Sample: 45 stroke survivors  Age: 66 ±10.0 years  Setting: two community-based rehabilitation centers | TUG, 10mWT, FTSST, EuroQoL Index and EuroQol-Visual Analog Scale. | a. Physical training  (exercises and group circuit class therapy). Duration: 2 hours daily, 5 days per week.  b. Leisure activities (preparing meals or cooking retraining, art, music and handicraft making) Duration: Once a week  c. Speech therapy  Duration: weekly | Significant improvement (P<0.02) was observed for TUG, FTSST, EuroQoL Index and EuroQoL-VAS scores. | |
| Chua et al. (2015) (65) | Singapore | To test a Variable Automated Speed and Sensing Treadmill (VASST) using a standard clinical protocol. | Pre and Post | Sample: 10 stroke patients  Mean Age: 55.5 ± 9.8 years  Setting: Outpatient rehabilitation clinics | 10mWT, 6mWT, BBS and FAC. | VASST training.  Duration: 12 sessions, 3 times a week consecutively for 4 weeks. Each session lasted for between 50 and 60 minutes.  . | Post-training gains in 10mWT (0.06m/s) and 6mWT (31.9m) were seen at week 2 (P <0.014, P <0.007, respectively).  Week 4: 9/10 gained in walking distance (+54.3m, SD = 30.9m, P = 0.005) while 7/10 subjects had improvements in self-selected gait speed on their 10mWT (+0.06m/s SD = 0.08m/s, P = 0.037). There were also significant gains in BBS score of 2.0 (SD = 0.29, P = 0.007).  Week 8: Gains in mean gait speed (+0.12m/s SD = 0.1, P < 0.01) and mean walking capacity (+56.0m, SD = 31.8, P =0.005) were recorded a month after cessation of training. | |
| Chee et al. (2014) (63) | Singapore | To retrain ambulation on  VAAST | Pre and Post | Sample: 10 chronic ambulatory stroke patients  Median age: 55 years  (38-71 years)  Setting: Tan  Tock Seng Hospital’s Center for Advanced Rehabilitation  Therapeutics | 10mWT, 6mWT, BBS, FAC | VASST training  Duration: 12 sessions, 3 times a week, 4 weeks | A significant improvement in the average walking speed (P <0.01) and walking distance (P <0.01) when compared with the baseline. | |
| Zainun & Ruslan (2019) (73) | Malaysia | To examiner the BAL EX home-based balance exercise therapy | Case Report | Sample: 1 stroke patient  Age: 60 years old  Setting: community | Balance, postural control | Bal Ex home based balance exercise | Outcomes after 10 months: almost 70% of participant’s balance difficulties and posture improved, and she was able to perform routine daily activities better than before. | |
| Cayco et al. (2019) (59) | Philippines | To compare the changes in motor outcomes after a PNF program across four new cases of older people with chronic stroke with different chronicity and disability severity. | Case Report | Sample: Four stroke patients  Age (mean): 64.75 (62-69) years  Setting: Outpatient clinic | UMCT, FTSST, Mini-BESTest, ABC, Limits of Stability (LoS) | Pelvic anterior elevation and posterior depression, LE flexion-adduction with knee flexion, LE extension-abduction with knee extension, rolling from supine to side lying, pelvic bridging stabilizing reversals in standing, one leg stance and resisted forward walking.  Duration: Each case received 18 one-hour sessions (3 times a week for 6 weeks) of individualized treatment. | Cases 2 and 3 showed a change of one level on their UMCT-extension scores. Cases 2, 3, and 4 also showed significant change in their FTSST scores. Cases 2, 3, and 4 showed significant changes on the Mini-BESTest. All cases also showed varied positive changes in LoS. Balance confidence for cases 3 and 4 increased, while there was a decrease in case 2. | |
| Cayco et al. (2017) (58) | Philippines | To describe the effects of a PNF program on the balance, strength, and  mobility of an older adult with chronic stroke. | Case Report | Sample: One stroke patient  Age: 69 years old  Setting: university-based  pro-bono outpatient physiotherapy clinic | LoS, Mini BESTest,  FTSST, Upright Motor Control test-extension (UMCT-E) | PNF-based therapy  Sessions were divided into resisted mat exercises and resisted walking training. Duration: 1 hour, 3 times a week, 6 weeks (18 treatment sessions). | All dimensions of LoS improved. Cumulative score on the Mini-BESTest increased significantly by 4 points. A clinically meaningful change in lower extremity strength with a 2.8-s difference on the FTSST, while knee extension strength specifically changed from weak to moderate on the UMCT-E. Clinically meaningful change was observed in fast gait velocity at 0.15 m/s, but not in self-selected gait velocity. | |
| Zainun et al. (2016) (74) | Malaysia | To test the feasibility of BAL EX FOOT for gait and balance rehabilitation | Case Report | Sample: 1 stroke patient  Age: Not mention  Setting: community | Balance, range of motion | Bal Ex Foot | In general, after using the shoes, the participant’s gait improved by almost 70% from baseline data and there was a reduction of external rotation of the lower left limb. In terms of balance, the participant reported that his balance improved a lot and felt comfortable. | |

| Observational Studies | | | | | | |
| --- | --- | --- | --- | --- | --- | --- |
| Author Citation | Country | Study Objective | Study Design | Setting and Participants | Instruments | Findings |
| Bower et al. (2019) (66) | Singapore, Australia | To investigate the predictive strength of gait and balance variables for evaluating post-stroke falls risk over 12 months following rehabilitation discharge. | Prospective Cohort | Sample: 56 /81 stroke patients (Singapore)  Age: 62.99 ±13.22 years  Setting: Inpatient rehabilitation facilities | Self-reported falls, TUG, Wii Balance Board (WBB), Step Test, Center of Pressure (CoP), 6mWT, gait speed, stride length, step width, gait speed variability, mediolateral and vertical pelvic displacement | Predictors: after adjusting for country, prior falls and assistance, significant predictors of falls were mediolateral pelvic displacement (IQR-OR = 7.85), stride length (IQR-OR = 4.23) and step length asymmetry (IQR-OR=1.37) |
| Jalayondeja et al. (2014) (64) | Thailand | To determine if the findings at month 1 could correctly identify stroke patients who fell in the 6 months post-stroke; and to describe the characteristics of fallers and non-fallers, and their courses of recovery. | Prospective Cohort | Sample: 97 stroke survivors  Age: 61.9 ±11.1 years  Setting: three stroke rehabilitation centers in Bangkok, Thailand. | Self-reported falls, BBS, BI, 10mWT, 2-min walk test, participation sub-score of Stroke Impact Scale, FES-S, Modified Thai MMSE, Fugl-Meyer, Modified Ashworth Scale (MAS), National Institute of Health Stroke Scale, BI | At the 6-month assessment, all functional outcome measures were statistically different between fallers and non-fallers (P < 0.05), except for ambulation tests (TUG and 10mWT). |

| Author Citation | Country | Study Objective | Study Design | | Setting and Participants | | Instruments | | Findings |
| --- | --- | --- | --- | --- | --- | --- | --- | --- | --- |
| Chaiwanichsiri et al. (2006) (62) | Thailand | To determine the incidence and risk factors of falls during inpatient stroke rehabilitation | Prospective Cohort | | Sample: 151 stroke patients  Mean Age: Fallers (63.8 ±10.8 years), Non-fallers (62.1 ±11.5 years)  Setting: Rehabilitation center | | Self-reported falls, BI, BBS, Scandinavian Stroke Scale, Thai Geriatric Depression Scale, Thai (MMSE), Snellen’s Chart, Clock Drawing Test, Copy of Drawing Test, Line Bisection Test, Cancellation Test | | BI and BBS of both groups were significantly different (P=0.013 and P=0.035) respectively and participants who had better function and balance fell more frequently. Patients with BI of less than 12 had more risk to fall and compared to the group with those with lower Barthel Index score, the risk increased about 3 times. |
| Chin et al. (2013) (76) | Singapore | To investigate the factors affecting the incidence of falls among individuals with stroke living in the community one year after discharge from a rehabilitation hospital | Cross Sectional | | Sample: 126 stroke survivors  Age (Median): 61 (interquartile range: 53-71) years  Setting: Community | | Self-reported falls, Fugl-Meyer Assessment, Functional Independence Measure (FIM) and BBS | | Factors associated with falls: longer length of hospital stay, lower BBS and lower-limb FM scores, and lower discharge FIM scores for the bladder and bowel management, transfer, mobility, communication, and social cognition domains (p < 0.05). The fallers were more likely to use walking equipment and required assistance with basic activities of daily living after discharge (p < 0.05). FIM scores revealed the transfer domain as the only significant independent factor for falls (OR: 0.78, 95% CI 0.62–0.99; P < 0.05). |
| Aziz et al. (2011) (57) | Malaysia | To assess the outcome of a multidisciplinary-based outpatient rehabilitation service for stroke patients living in the community. | | Longitudinal | | Sample: 68 stroke patients  Age: 62.4 ±12.4 years)  Setting: university hospital | | Modified Barthel Index (MBI), BBS | Multivariate analysis of progress over time for MBI demonstrated significant effect of intervention, F (3,7) =10.40, P=0.006. The BBS also had similar effect of the intervention, F (3,10) =5.53, P=0.017. |

| Qualitative Studies | | | | | |
| --- | --- | --- | --- | --- | --- |
| Author Citation | Country | Study Objective | Study  Design | Setting and Participants | Findings |
| Xu et al. (2019) (72) | Singapore | To investigate the perspectives of rehabilitation therapists on the implementation of fall  prevention programs and elicits recommendations to adapt the Stepping on program with stroke survivors. | Focus Group Discussion | Sample: 23 rehabilitation therapists (15 occupational  therapists; 8 physiotherapists)  Years of clinical experience: 9.3 ±5.5 years  Setting: Community | Three themes that emerged from the focus groups were limitations of existing falls prevention intervention for stroke clients, the need to adapt the Stepping On program to use with stroke clients and challenges in implementing fall prevention programs in the stroke context. A series of new components were suggested to be included as part of the program, including participation of family members and caregivers, and tailored community reintegration sessions. |
| Xu et al. (2019) (71) | Singapore | To explore participants’ experiences of self-perceived fall risk factors after stroke, common fall prevention strategies used, and challenges to community participation after a fall. | Semi-structured interviews | Sample: 17 (9 stroke survivors, 8 caregivers) respondents  Age of stroke survivors: 65 ±7.0 years  Setting: three community rehabilitation centers. | Common themes: self-perceived fall risk factors are divided into two: intrinsic and extrinsic.  Challenges: fear of falling, increased level of self-care, restricted mobility, and social participation.  Different themes: motivational factors in developing adaptive safety strategies, adaptive safety strategies, self-coping strategies, factors shaping the post-fall, experience, and protective safety strategies |

| Author Citation | Country | Study Objective | Study Design | Setting and Participants | Findings |
| --- | --- | --- | --- | --- | --- |
| Koh et al. (2014) (68) | Singapore | To explore what the obstacles are that deter patients from continuing stroke rehabilitation after  discharge from hospital in Singapore | Individual semi-structured telephone interviews | Sample: 58 stroke patients  Age: Continued with  rehabilitation (62.25 ±9.79 years), Did not continue with rehabilitation (66.46 ±12.01 years)  Setting: community | Despite the improvement in functional performance after inpatient rehabilitation, some patients were deterred from continuing rehabilitation as they did not feel the gain from their rehabilitation program. One participant commented that they did not see improvement after rehabilitation, the impaired body could still cause a fall and resulted in not being able to straighten the patient up. |
| Nordin et al. (2014) (70) | Malaysia | To explore the perception of rehabilitation professionals and people with stroke towards long term stroke  rehabilitation services and potential approaches to enable provision of these services | Focus Group Discussion | Sample: 15 rehabilitation professionals and 8 long term stroke survivors.  Age Range: Rehabilitation  Professionals (27-54 years), Stroke survivors (30-72 years)  Setting: three hospitals and one community stroke rehabilitation center | Participants also claimed that family members can be overprotective and that this had discouraged stroke patients from performing home exercises while other participants felt that the family of stroke patients had not given adequate support throughout the rehabilitation process. One stroke survivor quoted that whenever he does the exercises, his wife will be angry because she is afraid that by doing the exercises, he could fall. She is also worried who will take care of the stroke survivor if he does fall while doing the exercises. |
